# Postbiotics, Metabolic Signaling, and Cancer

**DOI:** 10.3390/molecules26061528

**Published:** 2021-03-11

**Authors:** Nikola Vrzáčková, Tomáš Ruml, Jaroslav Zelenka

**Affiliations:** Department of Biochemistry and Microbiology, University of Chemistry and Technology Prague, 16628 Prague, Czech Republic; Nikola.Vrzackova@vscht.cz (N.V.); Tomas.Ruml@vscht.cz (T.R.)

**Keywords:** microbiome, colorectal cancer, intestinal metabolome, GPR81, SCFA, functional food

## Abstract

Postbiotics are health-promoting microbial metabolites delivered as a functional food or a food supplement. They either directly influence signaling pathways of the body or indirectly manipulate metabolism and the composition of intestinal microflora. Cancer is the second leading cause of death worldwide and even though the prognosis of patients is improving, it is still poor in the substantial part of the cases. The preventable nature of cancer and the importance of a complex multi-level approach in anticancer therapy motivate the search for novel avenues of establishing the anticancer environment in the human body. This review summarizes the principal findings demonstrating the usefulness of both natural and synthetic sources of postbotics in the prevention and therapy of cancer. Specifically, the effects of crude cell-free supernatants, the short-chain fatty acid butyrate, lactic acid, hydrogen sulfide, and β-glucans are described. Contradictory roles of postbiotics in healthy and tumor tissues are highlighted. In conclusion, the application of postbiotics is an efficient complementary strategy to combat cancer.

## 1. Introduction

The gastrointestinal tract is colonized by a variety of different microorganisms, the so-called intestinal microbiome. This dynamic ecosystem is formed by a pool of 400–1000 adherent and non-adherent bacteria species [1], with approximately 1011 bacteria per gram of intestinal content [2]. Despite significant differences between the compositions of gut microbiomes in humans, their functions are very similar. They are responsible for the fermentation of indigestible food components into absorbable metabolites [3], detoxification of pollutants [4], and synthesis of a number of vitamins, mostly vitamin K and some water-soluble vitamins B [5], and they minimize contact with ingested bacteria, reduce the growth of pathogenic flora, and regulate the immune system [6]. The absorption of microbial metabolites into the circulation is responsible for the projection of its effects beyond the gut called the gut–body–brain axis due to the influence on the whole body signaling including the brain [7,8].

Any imbalance of gut microbiota, so-called dysbiosis, therefore, negatively influences the overall health of the host. While acute dysbiosis caused by food poisoning or antibiotic treatment is associated with temporary symptoms and does not necessarily need treatment with high-risk drugs [9], chronic dysbiosis has been linked to serious chronic diseases headed with autoimmune diseases, allergies, psychiatric disorders, obesity, and even cancer [10]. Therefore, the manipulation of the composition and the overall metabolism of intestinal microflora has been suggested as an effective preventive and therapeutic strategy to combat cancer and other diseases. Currently, there are four major types of such intervention: prebiotics, probiotics, synbiotics, and postbiotics. The definitions are as follows:

- Prebiotics are non-digestible food ingredients that beneficially affect the host by selectively stimulating the growth and/or activity of a limited number of bacteria in the colon, thus improving the host’s health.
- Probiotics are living microorganisms that, when administered in adequate amounts, confer a health benefit on the host.
- Synbiotics are combinations of prebiotics and probiotics that have beneficial effects on the gut microbiome.
- Postbiotics are substances released by or produced through the metabolic activity of the microorganisms which directly exert beneficial effects on the host [7].

While the research on probiotics has begun more than 100 years ago, postbiotics are a newcomer in the field of science, even though the beneficial effects of fermented food have been known for millennia [11]. Until recently, the studies have been focused on the effects of probiotics, mainly lactic acid-producing bacteria such as *Lactobacillus* or *Bifidobacterium*, and on prebiotics such as dietary fiber, human milk oligosaccharides, lactulose, and inulin derivatives. However, the increasing body of evidence suggests that the overall metabolism of the intestinal microflora is more important than the presence or absence of any particular microbial species. The postbiotics research was initiated by studies employing the cell-free supernatants (CFSs) from bacterial fermentation followed by primary microbial metabolites such as lactic acid and short-chain fatty acids (SCFA) [12,13,14,15,16]. However, there are numerous other interesting molecules including the odorous gas hydrogen sulfide (H_2_S) and a group of polysaccharides with mixed prebiotic/postbiotic properties called β-glucans. While several recent high-quality reviews covered the relationship between the intestinal microbiome and cancer [17,18,19,20,21,22], we focused on the available data on postbiotics and cancer with a special emphasis on metabolic signaling.

## 2. Metabolic Signaling in Cancer

Here, we hypothesize that an important component of the anti-cancer effects of postbiotics is the induction of cell response to metabolic and oxidative stress. As microbial metabolic waste products, postbiotics disturb the metabolic homeostasis of host cells, causing metabolic and oxidative stress. Lactate has been shown to stimulate mitochondrial production of reactive oxygen species (ROS) by increasing the NADH/NAD^+^ ratio and serving as a substrate of lactate oxidase [23]. Butyrate has also been shown to stimulate ROS levels, although the exact mechanism is unknown [24]. H_2_S covalently modifies sulfhydryl moieties of proteins and inhibits mitochondrial cytochrome c oxidase enhancing the ROS release [25]. Even β-glucans have been demonstrated to cause oxidative stress in cancer cells [26].

The adaptive response of the cells is driven by increased activity of stress signaling pathways, including, but not limited to, the energy sensor AMP-activated protein kinase (AMPK), the family of sirtuin deacetylases (SIRT1-7), the metabolic transcription coactivator PGC1α, the antioxidant transcription factor NFE2L2 (Nrf2), and the stimulation of specific survival processes including mitochondrial biogenesis and autophagy [23]. The enhanced stress signaling protects normal tissues against carcinogenesis by suppressing cell proliferation, mutagenesis, and tissue inflammation. Specifically, AMPK activity mimics the cancer-preventing effects of caloric restriction by enhancing autophagy and suppressing mTOR signaling [26]. It is also an activator of sirtuins and PGC1α that inhibit the cell cycle and nuclear factor kappa B (NFκB) signaling [27]. In addition, Nrf2-driven transcription stimulates the antioxidant, detoxification, and DNA repair capacity of the cells [23]. It is important to note that butyrate induces Nrf2 directly through its effect on histone acetylation [28,29].

**Figure 1:**
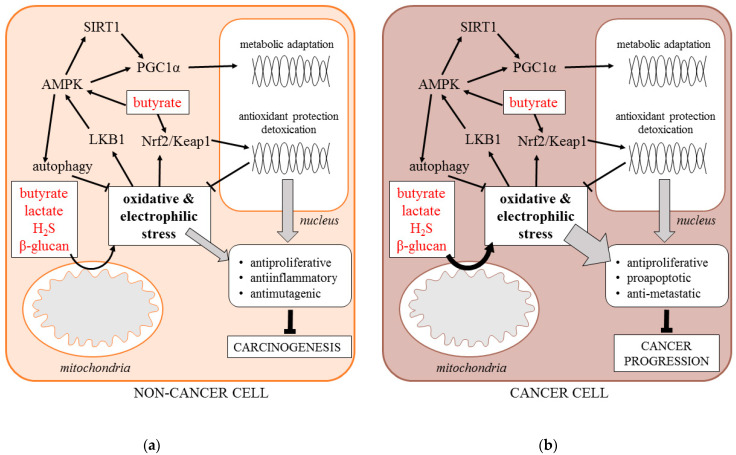
Signaling response of non-cancer (**a**) and cancer cell (**b**) to postbiotics (AMPK—5’ AMP-activated protein kinase, H_2_S—hydrogen sulfide, Keap1—Kelch Like ECH Associated Protein 1, LBK1—liver kinase B1, Nrf-2—Nuclear factor erythroid 2-related factor 2, PGC1α—Peroxisome proliferator-activated receptor gamma coactivator 1-alpha, SIRT1—Sirtuin 1).

On the other hand, the increased production of mitochondrial ROS accompanied by the enhanced stress signaling frequently occurs in cancer cells as a byproduct of metabolic transformation. Moreover, it is indispensable for the progression of the established malignant tumors due to its pro-survival effect [23,26]. However, such deregulated signaling may be overwhelmed by external metabolic stress, including postbiotics. Therefore, postbiotics also suppress the progression of established tumors by exaggerating the metabolic and oxidative stress, causing programmed cell death [23,24,25,26,27] (Figure 1). The suggested roles of postbiotics in stress response will be documented throughout the present review.

## 3. Cell-Free Supernatants

CFSs are typically prepared by centrifugation of microbial cultures followed by filtration. It was shown that these solutions have anti-inflammatory, antioxidant, antibacterial, anti-infectious, and anticancer effects [7]. The protective effects of CFSs were described for all the stages of tumorigenesis, including the initiation, growth, and metastatic dissemination.

In 2019, Bahmani et al. reported the suppressive effects of *Bifidobacterium bifidum* on the proliferation of human colon cancer cell line SW742 in vitro. It was demonstrated that even the CFSs from these bacteria can reduce the growth of tested cancer cell line [30]. In addition, Kim et al. showed the inhibition of cancer cells growth by CFS from *Bifidobacterium adolescentis SPM0212* using the three colon cancer cell lines Caco-2, HT-29, and SW-480 [31].

In 2012, Escamilla et al. published a study connecting CFS with cancer cell invasion. They used CFSs from two species: *Lactobacillus casei* and *Lactobacillus rhamnosus* GC, which were tested in vitro on human colorectal cancer cell line HCT-116. The main focus of this study was the effect of CFS on the ability of cancer cells to form metastases [12]. During the process of metastasis, there are two main steps: the induction of matrix metalloproteinases that degrade the components of extracellular matrix, such as collagen, fibronectin, and gelatin [32], and the loss of tight junction proteins, such as occludin, claudin, or zona occludens 1, 2, or 3, that exist between enterocytes [33]. It was shown that both CFSs reduced invasion of HCT-116 in vitro by decreasing the activity of metalloproteinases and increasing the level of zona occludens protein [12].

In 2020, a study was published linking the initiation of carcinogenesis with CFS application. CFS from *Lactobacillus plantarum* prevented tumor development in high-fat diet-fed C57BL/6-APC^Min/+^mice. This effect was achieved by enhancing the immune system, downregulating the inflammatory NF-κB and Wnt signaling pathways, and balancing the gut microbiota composition towards healthy control [34].

Finally, a supernatant from *Lactobacillus plantarum* could also reverse the resistance of colorectal cancer cells HT-29 and HCT-116 to 5-fluorouracil therapy by inactivating the Wnt/β-catenin signaling pathway [35].

## 4. Short-Chain Fatty Acids

The most studied postbiotics are SCFA, namely acetate, propionate, and butyrate. These molecules are products of dietary fiber fermentation by gut microorganisms, mainly *Faecalibacterium prausnitzii* and *Eubacterium rectale* [31]. SCFA contribute to 5–15% of the total caloric requirements of humans and the molar ratio of acetate:propionate:butyrate is approximately 60:20:20 [36,37].

The total production of intestinal SCFA is difficult to determine because 95% is taken up by colonocytes and metabolized [36]. Acetate and propionate are primarily transported to muscles and the liver, where they serve as substrates for mitochondrial oxidation. In contrast, butyrate is metabolized in situ by colonocytes [38], where it makes up to 70% of the energy demand [39]. Lower levels of intestinal SCFA were associated with the occurrence of advanced colorectal adenoma [40]. Since butyrate is the most-studied postbiotic, the next chapters will discuss in detail its role in cancer prevention and therapy.

### 4.1. Import to the Cells

As a small hydrophobic molecule, butyrate is able to diffuse through the membrane on the apical side of colonocytes [41]. In addition, two types of transporters also selectively import it: Monocarboxylate Transporter 1 (MCT1) and Sodium-coupled Monocarboxylate transporter 1 (SMCT1), both expressed on the apical side of the intestinal epithelium [42,43].

MCT1 employs a symport with H^+^ ions driven by a transmembrane gradient affecting the intracellular pH [44]. The presence of inflammation including inflammatory bowel disease or early stages of colorectal carcinogenesis is connected to the downregulation of MCT1 expression [45,46]. On the other hand, advanced stages of colorectal carcinoma (CRC) are associated with an upregulation of MCT1 expression [47] due to the intratumoral production and metabolism of lactate, another MCT1 substrate. Interestingly, physical activity, which is known to reduce the risk of CRC, increases MCT1 protein and its activity in muscle [48].

Butyrate transport through the SMCT1 is connected with a symport of one Na+ ion [49]. Similar to MCT1, factors that are responsible for the development of early colon tumorigenesis, for example, inflammation or obesity, downregulate the expression of SMCT1 [50,51,52]. SMCT1 is also silenced in thyroid, head and neck, breast, stomach, prostate, pancreas, and blood cancer [51,53]. A much less-described way how to transfer SCFA to the cell is by using the SCFA/HCO_3_^-^ exchange [54].

One of the transporters located on the apical side of the cell is the Breast Cancer Resistance Protein (BCRP). Butyrate is transported through BCRP coupled with ATP hydrolysis [55]. Interestingly, a malignant transformation of the colon epithelium is accompanied by a significant downregulation of BCRP and accumulation of butyrate in the cell [56]; however, in colorectal invasive cancer, this protein is upregulated and its level can be correlated with the ability to create metastases [57,58].

Considering the import of butyrate to the cell, it is also very important to mention the export [55]. A transporter located on the basolateral side that is responsible for the efflux of butyrate, Monocarboxylate transporter 4 (MCT4), is also connected with a symport of proton [59].

### 4.2. Mechanism of Action

#### 4.2.1. Metabolism

**Figure 2:**
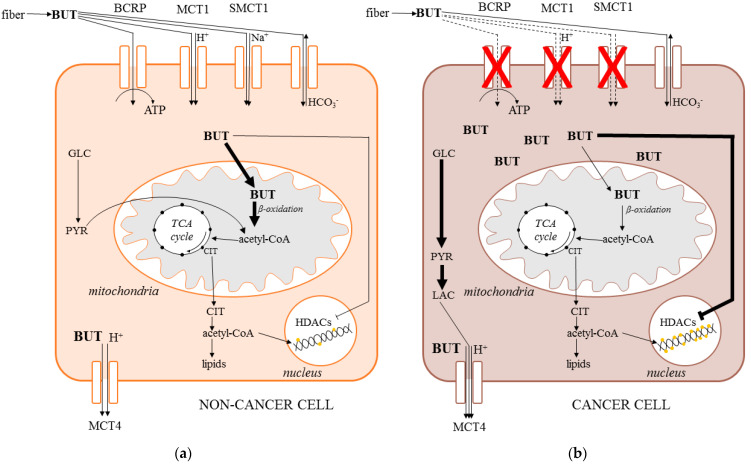
Import and metabolism of butyrate in non-cancer (**a**) and cancer cell (**b**) (BCRP—breast cancer resistance protein, BUT—butyrate, CIT—citrate, HDACs—histone deacetylases, MCT—monocarboxylate transporter, GLC—glucose, LAC—lactate, PYR—pyruvate, TCA—tricarboxylic acid cycle).

When transported to the cell, butyrate is quickly metabolized. In colonocytes, the roles of butyrate are very broad, including being the main source of energy, supporting growth and proliferation, protecting from apoptosis, inhibiting colorectal carcinogenesis, inflammation, and oxidative stress, and promoting angiogenesis [60]. In contrast, the role of butyrate in colon cancer cells is very different. Here, the butyrate supports differentiation, suppresses the proliferation, and evokes oxidative stress, leading to cell cycle arrest and apoptosis. Opposite roles of the butyrate in normal and cancer cells contribute to the phenomenon called “butyrate paradox” [60], which has not been explained until recently (Figure 2).

In a non-cancer cell, butyrate is metabolized by β-oxidation, accompanied by the utilization of acetyl-CoA in the Krebs cycle [61]. In cancer cells, the main source of energy is glucose, which is metabolized via glycolysis to lactate, a phenomenon called the Warburg effect, which is associated with the suppression of β-oxidation. Thus, molecules of butyrate accumulate in the cancer cell cytoplasm, migrating to the nucleus, where they act as inhibitors of histone deacetylases (HDACs). Under these conditions, the histones are acetylated and the solid structure of DNA becomes loose and accessible to the transcription factors, leading to the expression of different proteins, triggering oxidative stress and eventually apoptosis [62,63,64].

#### 4.2.2. Oxidative Stress

Oxidative stress is a result of the imbalance between the levels of prooxidative reactive oxygen and nitrogen species and the levels of antioxidant enzymes and scavengers that protect the cell against the oxidation of macromolecules [65].

A study by Abrahamse et al. showed that pretreatment of isolated distal colon cells with butyrate or acetate decreased the oxidative damage caused by hydrogen peroxide and stimulated the DNA repair and the antioxidant defense system. On the other hand, an increase of intracellular calcium induced by hydrogen peroxide was not changed by the presence of butyrate [14]. Therefore, butyrate may serve as a chemopreventive agent [66].

Similar results were obtained in the study of Rosignoli et al. dealing with freshly isolated human colonocytes and colon cancer cells. The H_2_O_2_-induced DNA damage was counteracted by butyrate and a mixture of SCFA, where butyrate was the most active component [67].

Other studies focused on the connection between antioxidant enzymes and SCFA. A study by Sauer et al. pointed to the ability of primary colon cells treated with butyrate to increase the expression of catalase and metallothionein while reducing the levels of cyclooxygenase-2, suggesting an antioxidant and anti-inflammatory effect. However, the levels of superoxide dismutase and glutathione-S-transferase were reduced [13]. In contrast, evidence of a butyrate-induced increase in glutathione-S-transferase levels in primary colonocytes and colorectal adenocarcinoma cells, but also premalignant adenoma, was reported [68,69,70].

#### 4.2.3. Autophagy

Autophagy (self-eating) is a complex pro-survival pathway triggered by nutrient depletion, starvation, or hypoxia. Proficient autophagy is indispensable for tumor progression. However, deregulated and excessive autophagy may cause autophagic cell death [71].

Butyrate was shown to be the inducer of autophagy in colorectal carcinoma cell lines through the aggravation of endoplasmic reticulum stress. Mechanistically, butyrate stimulated the liver kinase B1/AMP-activated protein kinase (LKB1/AMPK) signaling pathway [72,73].

#### 4.2.4. Invasion

The metastatic dissemination of tumors to distant sites is a multifactorial process estimated to be responsible for 90% of cancer deaths. An indispensable facilitator of metastasis is the ability of cancer cells to cleave the components of the extracellular matrix, which is provided by the matrix metalloproteinases (MMPs), a group of zinc-dependent endopeptidases upregulated in invasive tumors [32]. Another factor is the number of tight junctions between the cells in the primary tumor [74] and the levels of adhesion molecules such as CD44, which is capable of the cell–cell and cell–matrix interactions responsible for the colonization of distant sites [75,76].

In the study of Zeng and Briske-Anderson, the prolonged treatment of fibrosarcoma cells HT1080 with butyrate have shown the increased levels of MMP-2 and MMP-9 proteins but also increased the levels of tissue inhibitors of metalloproteinase-1 (TIMP), which altogether resulted in the decreased ability of these cells to create metastases [77].

Barshishat et al. focused on the levels of adhesion protein CD44 in the highly metastatic human colon cancer cell line HM7. They showed a significant decrease of CD44 after treatment with butyrate and a complete loss of HM7 ability to form metastases in the liver of nude mice [78]. Taken together, butyrate is capable of affecting the potential of cancer cells to metastasize even under in vivo conditions.

#### 4.2.5. Apoptosis

The main features of apoptosis are the condensation of chromatin, the fragmentation of nuclei followed by the appearance of the apoptotic bodies, the loss of inner mitochondrial membrane potential, the release of cytochrome c to the cytoplasm, and the activation of specific proteases—caspases [79].

Already in 1993, Hague et al. showed that physiological concentrations of sodium butyrate induce apoptosis, which was accompanied by a detachment of cultured cells from the surface to the medium. In some of the tested colonic cell lines, over 80% of the cells were apoptotic with internucleosomal DNA fragmentation. The percentage of apoptotic cells was more significant for carcinoma cells compared to the adenoma cell lines [16,80].

The study of Chirakkal et al. investigated the mechanism of cell death after the treatment with butyrate in three colorectal cell lines. BAK pro-apoptotic protein levels were shown to be significantly increased, while Bcl-like anti-apoptotic protein levels were decreased. The study indicated that BAK is upregulated at the transcriptional level through increased binding of transcriptional factors to the promoter due to the typical inhibition of HDACs, leading to a loose DNA structure [81].

### 4.3. Tributyrin

Tributyrin, a triacylglycerol containing three molecules of butyrate, was tested as a postbiotic agent to avoid the disgusting odor and short half-life of free butyrate. Several in vitro studies on different cancer cell lines showed that tributyrin induces apoptosis at millimolar levels [82,83,84,85,86,87,88]. More importantly, the treatment with tributyrin also induced apoptosis in some in vivo studies. Giermasz et al. showed that the systematic administration of tributyrin significantly retarded the growth of B16F10 melanoma in mice [85]. This effect was also observed in another in vivo study on mice with transplanted prostate cancer cell lines [86]. These results motivated phase I clinical trials for tributyrin tolerability in cancer patients, which proved good tolerance and promising effects on the disease progression [87]. In addition, the preventive effects of tributyrin also deserve attention. The treatment with tributyrin reduced the frequency of preneoplastic lesions in a rat model of chemical hepatocarcinogenesis [88]. Tributyrin feeding also protected against intestinal injury and inflammation in various animal models, both phenomena being well-known contributors to intestinal carcinogenesis [89,90].

Taken together, tributyrin is a candidate chemopreventive drug triggering the cancer cell apoptosis, modulating the histone acetylation, and having a longer half-life compared with the butyrate.

## 5. Lactic Acid

### 5.1. Import to the Cell

Lactate, the anion of lactic acid, is accumulated in the cells by two main classes of transporters: a group of monocarboxylate transporters (MCTs) and a group of low-affinity sodium-coupled monocarboxylate transporters (SMCT2). The MCT family has 14 members with MCT1-4 being specific for the lactate, pyruvate, and ketone bodies in a proton-dependent manner [91,92].

MCTs are highly expressed especially in muscles, liver, kidney, intestine, and brain, but also in tumor tissue. MCT1 is a high-affinity, low-capacity preferential importer of lactate, while MCT4 is a low-affinity, high-capacity preferential exporter of lactate. Both MCT1 and MCT4 are dominant forms of MCTs, and are highly expressed in tumors [93].

The SMCT2 class contains only two members abbreviated SLC5A8 and SLC5A12. SLC5A8 is selective not only for lactate but also for SCFA and nicotinate [94]. SMCT2 is localized in only three tissues: kidney, small intestine, and skeletal muscles. The available data suggest that the main role of SMCT2 is the uptake of lactate from dietary sources [95].

### 5.2. Lactate Signaling

Lactate is a natural, high-affinity substrate, for the G protein-coupled surface receptor called GPR81. GPR81 is highly expressed in the adipose tissue and its pleiotropic signaling roles were also demonstrated in skeletal muscle, immune cells, and the central nervous system [96]. GPR81 is also upregulated in tumor tissue, and its crucial role in disease progression, metabolic flexibility, and immune evasion was demonstrated [97,98,99,100,101].

Another mediator of lactate signaling is the elevation of ROS production in mitochondria upon lactate exposure [102,103]. Lactate-derived ROS alone and in combination with GPR81 signaling is responsible for the stimulation of the stress response signaling including the AMPK, PGC1α, and mitochondrial biogenesis. The elevated stress response signaling is associated with a cytoprotective phenotype called mitohormesis [103,104].

### 5.3. Dual Role of Lactate in Cancer

The role of lactate, MCTs, and GPR81 in the tumor microenvironment is predominantly driven by the Warburg effect, i.e., enhanced glycolysis and lactate secretion of cancer cells even at normoxia, resulting in the tumor lactate levels reaching 10–20 mmol · L^−1^, while the normal concentration is around 1–2 mmol · L^−1^ [105].

The endogenously produced lactate in the established tumors has been identified as a driver of angiogenesis, immune evasion, metastasis, and resistance to therapy [106]. On the other hand, lactate delivered by postbiotic supplementation may serve as an inhibitor of carcinogenesis through the maintenance of the epithelial integrity and downregulation of the tissue inflammation [11,107,108,109,110,111].

### 5.4. Cancer Prevention with Fermented Dairy Products

Even though there is significant evidence regarding the chemopreventive properties of fermented food, especially dairy products, these studies are rather phenomenological without identification of the active compound(s) responsible for the effect. On the other hand, the main microbial components of fermented dairy products are lactic acid-producing bacteria (LAB). It is well known that LAB convert lactose in dairy products to lactic acid. There is about 0.9% of lactic acid in yogurts and 2% in kefirs. Food with such health-promoting properties is termed “functional food.” Based on the available data, it is likely that lactic acid is involved in the beneficial effects of fermented food.

In 2008, a large prospective study of the effect of fermented milk on 82,002 Swedish women and men was published. This study was focused on bladder cancer because most metabolites are excreted through the urinary bladder, providing a visible effect of orally administered dairy products. The results suggest that a high intake of cultured milk may reduce the risk of developing bladder cancer [112]. The results were later confirmed in a meta-analysis of available studies [113]. Other studies have shown that regular consumption of yogurt but not milk decreases the incidence of colorectal cancer in Europe and the US [114,115,116]. A higher adolescent dairy intake was associated with lower rectal and advanced adenoma risk later in life in a large cohort of nurses [117]. A regular yogurt intake versus a rare intake was associated with decreased odds of hyperplastic polyps and adenomatous polyps in the colon [118]. Taken together, postbiotic lactic acid may serve as a prevention of cancer, but further studies are needed to distinguish its effects from the other components of dairy products.

## 6. Hydrogen Sulfide

Hydrogen sulfide (H_2_S) is a colorless, flammable, and water-soluble gas with a very specific smell of rotten eggs [119]. For decades, H_2_S was perceived only as a toxic pollutant with a negative effect on the environment. However, recent investigations demonstrated that it is enzymatically produced in the human body serving as a gaseous hormone [120].

There are two main pathways of H_2_S production: either the endogenous pathway driven by the enzymes of human cells or the microbial pathway localized in the gut microbiota. The precursors of H_2_S synthesis are mainly the sulfur amino acids and the free sulfate. H_2_S produced in the gut should be considered a candidate postbiotic [121]. The production of H_2_S was described in the bacterial genera *Fusarium, Clostridium, Salmonella*, *Escherichia*, *Klebsiella*, *Streptococcus*, *Desulfovibrio*, *Enterobacter* and others [122].

### 6.1. Biological Effects

**Figure 3:**
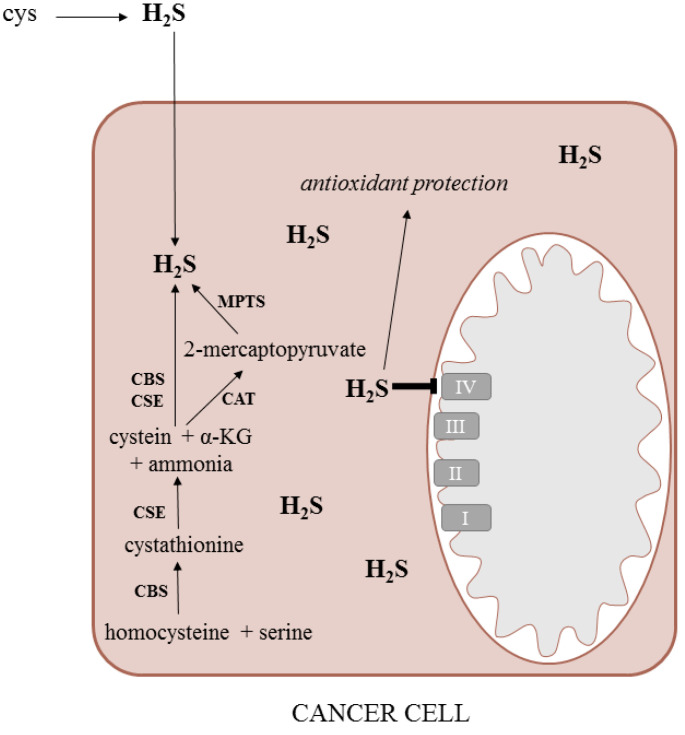
Synthesis and metabolism of H_2_S in the cancer cell (α-KG—alpha-ketoglutarate, H_2_S—hydrogen sulfide, CAT—cysteine: 2-oxoglutarate aminotransferase, CBS—cystathionine beta-synthase, CSE—cystathionine gamma-lyase, cys—cysteine, MPTS—3-mercaptopyruvate sulfurtransferase).

H_2_S possesses either a cytoprotective or a cytotoxic effect depending on its concentration and the cell type. At low concentrations, it serves as an antioxidant neutralizing various reactive oxygen species and acting additively to the known antioxidants, such as N-acetylcysteine, superoxide dismutase, or glutathione [123]. At high concentrations, H_2_S binds to the cytochrome c oxidase blocking the electron transport chain and diminishing the synthesis of ATP [115] (Figure 3). It can also initiate the expression of different proapoptotic genes and trigger apoptosis [124].

H_2_S acts mainly through the regulation of mitochondrial bioenergetics including the activation of potassium channels [125], stimulation of signaling kinases [126], and inhibition of phosphodiesterases [127].

### 6.2. Role in Cancer

#### 6.2.1. Colon Cancer

Due to the sulfate-reducing bacteria, the concentration of H_2_S in the colon reaches approximately 0.3–3.4 mmol · L^−1^ [128]. A high dose of H_2_S causes a decrease in the proliferation and the induction of the G_0_/G_1_ blockage of the cell cycle resulting, in an overall antimitotic effect. It can also induce autophagy and inhibit the invasion and migration of cancer cells [129]. Its effects, therefore, resemble SCFA.

On the other hand, it was demonstrated that silencing or inhibition of the endogenous enzymes producing H_2_S, often upregulated in tumors, leads to the suppression of the bioenergetics resulting in the reduction of the tumor growth in vivo [130]. In addition, the low doses of exogenous donors of hydrogen sulfide stimulate cell migration via the activation of Akt/PI3K [131].

In 2016, Ianaro et al. summarized the chemopreventive effect of H_2_S-releasing non-steroidal anti-inflammatory drug (NSAID) ATB-346. They used the aberrant crypt foci (ACF) model of colon cancer. This model is based on the creation of ACF after the administration of carcinogen and untreated lesions are then developing into the tumors. They demonstrated a synergy between NSAID and H_2_S and the superiority of the combined approach over H_2_S or NSAID alone in terms of tolerability and efficiency [132].

#### 6.2.2. Other Tumors

H_2_S produced by bacteria freely enters the cells because of its lipophilicity and small size [133]. In 2015, Cakmak published a study pointing at the connection between gut microbiota-produced hydrogen sulfide and Parkinson’s disease. He proved that a lower level of the gut bacteria *Prevotella* negatively affects the progression of Parkinson’s disease. This was probably due to the gut–brain axis [134]. Thus, this study shows that as a postbiotic, hydrogen sulfide can affect different tissues, not only the bowel, suggesting that this gas could also affect other types of cancer.

The sign of impaired H_2_S metabolism is the accumulation of metabolic byproducts thiosulfate or sulfite. These metabolites are excreted via urine, and higher levels are associated with prostate cancer. There is also a positive correlation between the level of thiosulfate and the tumor volume [119,135].

In contrast, Liu et al. summarized the effects of different H_2_S-releasing donors in the prostate cancer cell line PC-3. They demonstrated that sodium sulfide is a rapidly releasing donor of H_2_S that significantly decreases cell viability, reduces the rate of growth, and decreases cell clonogenicity [136,137]. Diallyl disulfide and diallyl trisulfide, the sulfide-based metabolites of garlic, compromise cell survival and induce the apoptosis in PC-3 line [138,139]. In addition, sulforaphane from the cruciferous vegetable (broccoli) also triggers apoptosis and reduces the growth of tumors by acting as an antiproliferative agent [140]. In general, numerous other exogenous H_2_S donors administered in relatively high doses manifested anticancer effects in vitro and in vivo [141,142].

Therefore, H_2_S is a postbiotics acting as a double edge sword. While the low levels are cytoprotective in both normal tissue and tumors, the very high levels cause tissue damage, which may be turned into anticancer therapy under certain conditions.

## 7. β-Glucans

The term β-glucans is used for a group of the glucose polymers consisting of a (1→3)-β-D-glucopyranosyl chain with randomly dispersed β-D-glucopyranosyl units attached by a (1→6)-β bond [143]. As a component of dietary fiber, β-glucans have been found in a variety of plants and microorganisms, including oat, barley, medicinal mushrooms, seaweed, some bacteria, and also yeasts, where they represent an important component of the cell walls [144,145]. β-glucans were identified as biological response modifiers stimulating the immune system to fight cancer 60 years ago [146].

### 7.1. Forms of β-Glucans

There are two main forms of β-glucans—the particulate and the soluble. The particulate β-glucans are administered orally [147]. They are ingested by gastrointestinal macrophages and transported to the spleen and the bone marrow. Because of the absence of glucanase, the macrophages metabolize β-glucans through an oxidative pathway. The treatment with β-glucans stimulates the macrophages to secrete the pro-inflammatory cytokines such as TNFα (tumor necrosis factor α) or IL-6 (interleukin-6) [148].

Soluble β-glucans are formed by a triple helix of glucose polymer. The helices have a β-(1–3)-glucan backbones containing β-(1–6)-linked β-(1–3) branches. This form of β-glucans is soluble in water, easy to be purified, and the molecules have an intermediate size [143]. The soluble β-glucans may be administered intravenously and then metabolized by macrophages [149].

### 7.2. Mechanism of Action

Antibody therapy is effective for patients with a high expression of specific target antigens on the cancer cell surface. C3b and iC3b are the components of the complement-dependent cytotoxic system that contributes significantly to tumor regression. These two components connected with the complement activation in the presence of natural or intravenously injected anti-tumor antibodies are capable of binding to the complement receptor CR3 on neutrophils, macrophages, and natural killer cells triggering the phagocytosis and cytotoxic degranulation. Unfortunately, this reaction is hindered by the absence of a non-specific co-stimulation with microbial biopolymers, normally present during the antibacterial response. However, the presence of β-glucans may serve as the co-stimulator triggering the tumoricidal activity of the innate immune system [150,151].

**Figure 4:**
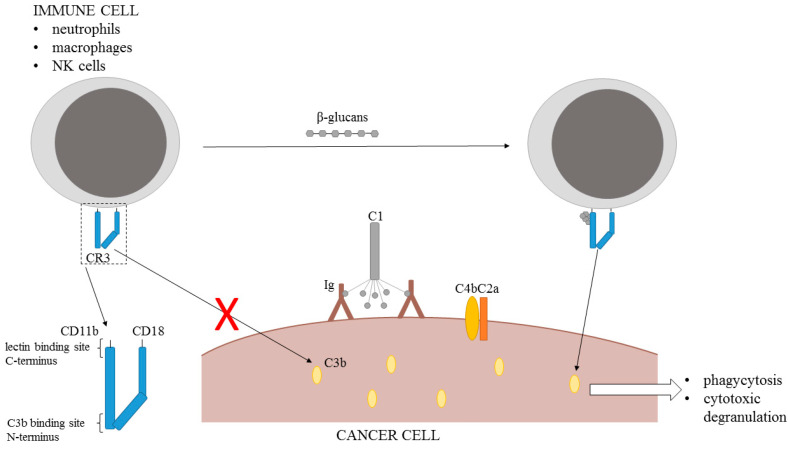
Mechanism of β-glucans action (CD—complement domain, CR—complement receptor, Ig—immunoglobulin).

CR3 receptor can recognize iC3 via the CD11b I-domain binding site on the N-terminus and the specific microbial polysaccharides via a lectin site located on the C-terminus. β-glucans bind to the lectin site, thus priming the immune cells to kill iC3b-coated tumor cells [152] (Figure 4).

A number of in vitro and in vivo studies pointed to this feature of β-glucans [143,146,151,153]. It has been demonstrated that β-glucans must be administered orally because intravenously administered glucans are rapidly scavenged by CR3 blood granulocytes and liver Kupffer cells, and only a small amount of glucans reaches the tissue macrophages that could migrate into tumors [154]. The study of Hong et al., using five mice tumor models, showed that tumor regression and animal survival were significantly enhanced in a group of mice receiving the combination therapy of monoclonal antitumor antibodies and β-glucans [151]. The therapy was successful only upon the natural antibody administration in the case of a mouse model with inherited severe combined immunodeficiency. Moreover, the therapy failed in C3-deficient mice. On the other hand, the study of Yang et al. discovered that β-glucans are effective even as a monotherapy in the presence of naturally occurring antitumor antibodies [155].

While β-glucans may also serve as prebiotics with anti-inflammatory properties [155,156], their direct immunity-stimulating postbiotic role will gain further attention due to the rising importance of cancer immunotherapy.

## 8. Conclusions

Postbiotics represent an emerging concept appreciating the importance of microbial metabolites in the maintenance of health. Butyrate, the prototypical SCFA, derived from the intestinal metabolism of fiber or supplemented as tributyrin inhibits carcinogenesis and selectively induces apoptosis in tumor cells. Lactate produced by LAB in fermented food or the gut serves as a signaling molecule in the host. It may inhibit early carcinogenesis by its anti-inflammatory and mitohormetic effects. However, the endogenously produced lactate is an important contributor to cancer progression. Therefore, LAB-fermented food is an important part of a cancer-preventing diet, but the application of lactate in the anti-cancer therapy is unlikely. Hydrogen sulfide is also a double-edged sword in the fight against cancer. While its endogenous moderate production is indispensable for cancer cell survival, the consumption of high-dose H_2_S donors, especially those of plant origin, is an emerging anticancer strategy. The broad family of β-glucans is well known for its immunomodulatory properties. The accumulating body of evidence demonstrates the utility of β-glucans co-treatment during anticancer immunotherapy. Taken together, the molecules inspired by microbiota-derived functional metabolites represent a novel and promising class of remedies applicable in cancer prevention and treatment.

## Data Availability

Not applicable.
